# Engineered *Escherichia coli* as a microbial cell factory for intracellular protein delivery: strains, vectors, mechanisms, and therapeutic applications

**DOI:** 10.1186/s12934-026-02944-9

**Published:** 2026-02-26

**Authors:** Sonia Dangi, Dek Shen Liew, Vetriselvan Subramaniyan, Kumaran Narayanan

**Affiliations:** 1 Jeffrey Cheah School of Medicine and Health Sciences, Monash University Malaysia, Bandar Sunway, 47500 Subang Jaya, Selangor Darul Ehsan Malaysia; 2 Department of Medical Sciences, Sunway University, Bandar Sunway, 47500 Petaling Jaya, Selangor Darul Ehsan Malaysia

**Keywords:** Genetically modified *E. coli*, Targeted drug delivery, Synthetic biology, Versatile vectors

## Abstract

*Escherichia coli* (*E. coli)* has emerged as a promising vector of therapeutic proteins into target cells due to its high fidelity to genetic manipulations, short generation times, and well-known molecular pathways. Over the years, the use of *E. coli* as a delivery vector has been explored in various mechanisms. This review aims to discuss the mechanisms through which *E. coli* can express and transport therapeutic proteins to target cells. Various delivery systems have been developed using *E. coli*, starting from the simple plasmid vectors, to outer membrane vesicles and, in some cases, live bacteria itself which can transport proteins into cells. These *E. coli* based systems are of immense potential in targeted drug delivery and therapeutic applications and have made *E. coli* to lead in novel biotechnological developments. However, there are still many challenges, concerning the improvement of the safety and efficacy of *E. coli* for protein delivery into cells, especially in regards to delivery efficiency and directional control in a real biological environment. Most of these challenges have been solved by the recent developments in synthetic biology, genetic engineering and *E. coli* is gradually becoming a versatile vector for protein therapeutic delivery into cells.

## Introduction

Intracellular delivery of functional proteins has emerged as an effective technique for therapeutic intervention, cellular engineering, and mechanistic research in mammalian systems [1]. Compared to nucleic acid-based techniques, direct protein delivery allows for quick and transitory manipulation of cellular activities while avoiding the dangers associated with genome integration, mutagenesis, and uncontrolled expression [2–4].

Despite the successful delivery of around 100 proteins in animal models, only some of them have entered in clinical trials [5, 6]. However, the efficient delivery of large functional proteins across the cell membrane remains a major challenge due to the several biological and technical barriers such as protein’s hydrophilic nature, membrane permeability, endosomal escape, and intracellular stability [7, 8].

Bacteria have lately acquired popularity as live carriers for delivery [9, 10]. They can have natural interactions with host cells. They are also genetically adaptable and capable of producing and disseminating complex macromolecules [11–13]. For example *E. coli*,* E.coli* is a well-known and versatile bacterial host in biotechnology [14]. It develops swiftly, has a diverse set of genetic tools, and performs well with synthetic biology procedures [11, 15]. Other examples includes commonly used pathogenic bacteria, such as *Salmonella* and *Listeria monocytogenes*, have been often employed for intracellular delivery [16, 17]. However, recent discoveries enable the safe delivery of functional proteins into mammalian cells [18]. This is achievable because of specially engineered non-pathogenic *E. coli* strains, like *E. coli* K-12 and *E. coli* Nissle 1917 [19].

A growing number of studies have been published that describe various engineering strategies for delivering genes and proteins intracellularly via *E. coli*, including tailored expression vectors, autotransporters, secretion pathways, Type III secretion systems [20], invasion proteins, cell-penetrating peptide fusions, and outer membrane vesicles [1, 19, 21]. These developments have resulted in the growth of *E. coli* applications into cancer therapy, immunological regulation, metabolic illness treatment, and intracellular enzyme replacement [16, 22, 23]. Despite the high speed of progress, the literature remains fragmented, with no single comprehensive assessment of vector engineering, intracellular absorption processes, and delivery techniques in the context of *E.coli*.

As a result, the purpose of this study is to give a comprehensive and critical assessment of the current techniques to engineering *E. coli* for intracellular protein delivery and expression. We talk about vector systems, secretion and absorption processes, distribution platforms including outer membrane vesicles and probiotic strains, and the new uses for these tailored systems. Furthermore, we emphasize the limits, safety concerns, and future potential for improving *E. coli* as a modular and therapeutically relevant intracellular protein delivery platform.

## Evolution of *E.coli* as an expression and delivery vector

*E. coli’s* development as an expression and delivery vector spans over four decades [14, 24]. *E. coli* emerged as a dependable protein expression host in 1978, with the effective recombinant synthesis of human insulin [25, 26]. During the 1990s and 2000s, engineered strains including BL21(DE3), Rosetta (1996), and SHuffle (2012) enhanced expression efficiency, codon use compatibility, and disulfide bond formation [24, 27, 28]. By the 2010s, breakthroughs in synthetic biology have enabled *E. coli* to transition from expression to intracellular delivery, including engineered Type III secretion systems (2010–2013), Type VI-based export platforms, and outer membrane vesicle (OMV)-mediated delivery (2014 onwards) [20, 21, 29, 30].

**Fig. 1:**
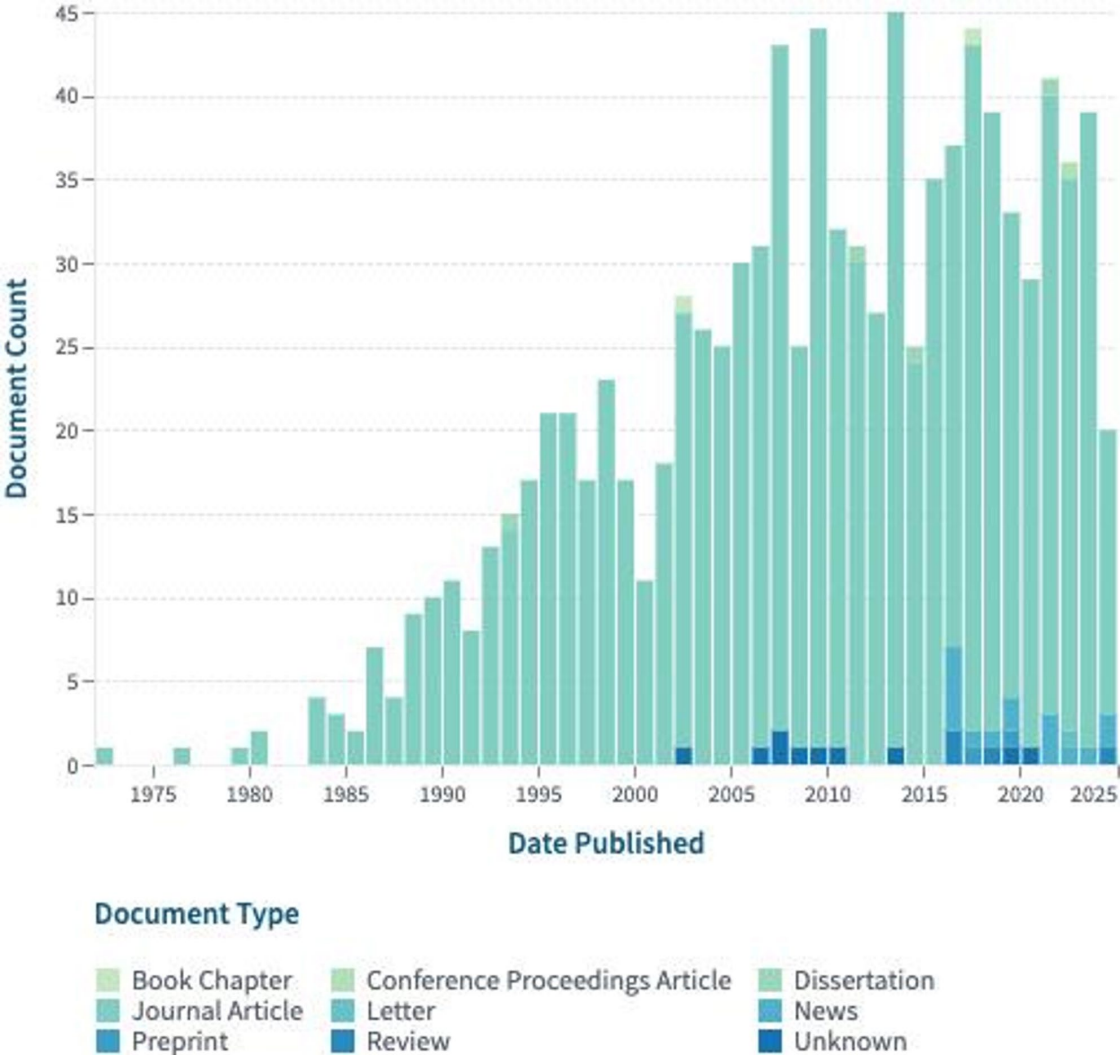
Overview of scholarly contributions in *E. coli* as a vector development (LENS.ORG). Figure depicts the evolution of research on *E. coli* as a vector for recombinant protein expression, gene delivery and protein delivery, reflects the growing interest in this subject area over time and provides an overview of their publishing history

*E. coli* has been proposed as a robust tool for gene cloning and expression, due to its fast growth [31], ease of transformation with exogenous DNA, and high efficiency of recombinant protein production [32]. In Fig. 1, a schematic presentation of the *E. coli* research with regard to the protein expression and vector development throughout the years is shown. Therefore, *E. coli* is a good model organism for the construction of versatile and potent vectors for biotechnological and medical purposes owing to its genetic manipulability and strong protein expression [33].

## Engineering *E. coli* for protein expression

Expression vectors represent the heart of engineered *E. coli* platforms for intracellular protein delivery, given that they are used to determine the level, timing, localization, and stability of the cargo protein [34]. Contemporary vector systems provide extensive flexibility via a tuneable copy number, promoter strength, secretion signals, and fusion tags, making it possible to provide precision control over heterologous protein expression suited to delivery applications [35, 36].

## Expression plasmids in *E. coli*

When discussing *E. coli* plasmids, *E. coli* vectors are valuable assets in the field of molecular biology that are used extensively in a variety of biotechnological applications [37]. Plasmid copy number has a profound effect on both expression yield and metabolic burden. High-copy plasmids, like those based on pUC or pET vectors, allow for high yields of protein (> 100 copies/cell) but may lead to instability or growth retardation [38, 39]. Among them, pET vector series is widely applied for recombinant protein expression and contributes to the structure-based research of biology, drugs, and biopharmaceuticals production [33]. The second is pUC19, which is one of the most popular cloning vectors, which has been applied to gene cloning, sequencing, and the construction of recombinant DNA libraries [40]. Conversely, low-copy plasmids such as p15A, pSC101, and F-origin provide higher stability and lower toxicity, allowing their use in balanced protein production in delivery systems [41].

There are also some classic vectors, such as pBR322 [42], which can be used for gene cloning, mutagenesis, and the study of antibiotic resistance. On the other hand, pBAD series helps in the regulation of gene expression and is useful in the research of gene regulation, networks, and pathways [43]. Moreover, there is a special plasmid such as pGLO, which is a plasmid that is commonly used in molecular biology to express the green fluorescent protein (GFP) that was initially derived from *Aequorea victoria*, a jellyfish species [44]. This plasmid contains several key features, including the GFP gene, an ampicillin resistance gene (which can be used to select for bacteria into which the plasmid has been successfully transformed), and the pGLO vector is utilised in educational and scientific studies, particularly in the investigation of gene and protein localization [45]. This shows how various kinds of plasmid-based *E. coli* vectors are employed in molecular biology studies and how they can be used to solve number of biological problems and challenges [46, 47].

## Synthetic gene circuits for controlled intracellular protein delivery

Advances in synthetic biology are increasingly enabling *E. coli* to serve as programmable microbial cell factories for targeted intracellular protein delivery [41]. A main determinant of successful delivery is the ability to precisely control when and where therapeutic proteins are being expressed, secreted, and released [19, 48]. Recent years have seen significant advances in gene circuit engineering, yielding sophisticated regulation methods that can match bacterial behaviour to clinically relevant microenvironments, thus increasing specificity, efficacy, and safety [49].

The design of hypoxia-inducible regulatory modules represents one area of significant development, as it aligns well with the pathological features of solid tumors [50]. Such design integrates promoters and transcriptional regulators that have been developed through exploration of responses to low-oxygen conditions and allows the engineered *E. coli* to selectively activate therapeutic expression in the hypoxic core of the tumor, while remaining inactive in normoxic tissues [50, 51], these circuits avoid dependence on exogenous inducers and highlight how disease-associated physiological cues can be leveraged as natural control signals [52]. The sensor repertoire is expanding by exploiting similar strategies for sensing acidic pH, reactive oxygen species, bile salts, or markers of inflammation, thereby enabling delivery that is increasingly context-specific [53].

In parallel, programmed lysis systems have emerged as powerful tools for intracellular payload release [54]. Engineered autolysis modules enable bacteria to accumulate therapeutic proteins intracellularly and then release them upon entry or replication within target cells [55]. These circuits often include feedback regulation or environmental sensing to ensure that the lysis only occurs after the bacteria reach an appropriate niche [56]. Besides enabling efficient cargo release, programmed lysis further contributes to safety by limiting bacterial persistence and providing built-in mechanisms for clearance [7].

A further layer of control involves tuning secretion-system activity, especially through regulated expression of master transcriptional regulators associated with T3SS [57]. Such modulation allows for dynamic adjustment of secretion efficiency, effector protein specificity, and timing of translocation into host cells [58]. The integration of regulation at the level of T3SS into synthetic circuits allows secretion to be coupled to host-derived cues or states of bacterial stress, enhancing the responsiveness and adaptability of the delivery system [58, 59].

These advances collectively demonstrate the development from simple, chemically induced constructs to complex, environmentally responsive circuits of genes that bestow a higher level of decision-making capacity on *E. coli* [60]. These systems are increasingly modular, predictive, and biocompatible, they greatly enhance the potential for using reengineered microbes as next-generation platforms for targeted intracellular protein delivery and therapeutic intervention [61]. Further development will need to be paired with computational design frameworks, optimization of the chassis, and rigorous safety engineering to ensure dependable performance in a wide range of in vivo applications.

## Engineered secretion systems

Engineered secretion systems allow *E. coli* to export heterologous proteins across the inner and outer membranes, improving their accessibility for subsequent delivery into host cells [15]. Autotransporters, especially AIDA-I, Ag43, and members of the β-barrel autotransporter family, have been extensively repurposed for display or secretion of proteins at the bacterial surface [62]. These systems are composed of an N-terminal passenger domain fused to a C-terminal translocator β-domain promoting export across the outer membrane [63]. By replacing the native passenger domain with therapeutic or reporter proteins, autotransporters enable efficient export and surface anchoring and, in some instances, release into the extracellular milieu [64].

In parallel, the Sec and Tat pathways remain foundational for directing proteins across the inner membrane [65]. The Sec system is responsible for the translocation of unfolded polypeptides and is widely utilized for the high-yield secretion of enzymes and signalling proteins [66]. However, the Tat pathway can uniquely enable the export of fully folded proteins and is well-suited for the transport of redox-sensitive or cofactor-bound proteins [15]. Downstream, *E. coli* T1SS, such as the HlyA-dependent pathway, allows for a single-step transport from the cytoplasm to the extracellular environment [67]. The adaptation of these engineered T1SS modules has been utilized to secrete a wide range of heterologous proteins including cytokines, nanobodies, and reporter enzymes, thus facilitating a more efficient delivery from host cells, especially upon combination with invasive entry or vesicle-mediated mechanisms [15, 68]. These secretion platforms together represent a versatile toolbox toward customizing how *E. coli* presents and/or exports proteins destined for intracellular delivery.

## Fusion tags for enhanced protein expression and delivery

Fusion tags strongly contribute to enhancing expression, stability, solubility, and intracellular trafficking of recombinant proteins produced in *E. coli* [69]. Such tags, however, become particularly important when engineering *E. coli* as a protein-delivery vehicle due to their previously mentioned enhancement of proper folding, protection from degradation, and facilitation of targeting to cellular compartments.

Solubility-enhancing tags such as Maltose-binding protein **(**MBP) and Small ubiquitin-like modifier (SUMO) help proteins correctly fold in the bacterial cytoplasm, increasing yields of functional products suitable for downstream delivery [70, 71]. Small affinity tags, like His₆, FLAG, and Strep-tag II, are commonly used to purify proteins prior to packaging into OMVs or coupling with delivery scaffolds [72]. Tags with secretion roles, such as Pectate lyase B leader sequence (PelB) or Outer membrane protein A (OmpA) signal peptides, can direct proteins to the periplasm or outer membrane, improving accessibility for OMV loading or surface display [73–75].

Furthermore, specific fusion partners have the additional ability to directly promote uptake into mammalian cells. For instance, Cell-penetrating peptide (CPPs) like Transactivator of transcription (TAT) or penetratin mediate non-endocytic translocation, whereas PTDs, when fused to cargo proteins expressed in *E. coli*, can strongly improve the efficiency of intracellular delivery [76, 77]. These functional tags connect the expression platform to the delivery mechanism and thus are an integral part of the bacteria-based delivery systems. Overall, fusion tags are not just expression tools but significant engineering elements impacting folding, secretion, targeting, and finally the efficiency in protein delivery from *E. coli* into host cells [78, 79].

## Mechanisms of intracellular entry: endocytic and non-endocytic pathways

### Endocytosis-based uptake

**Fig. 2:**
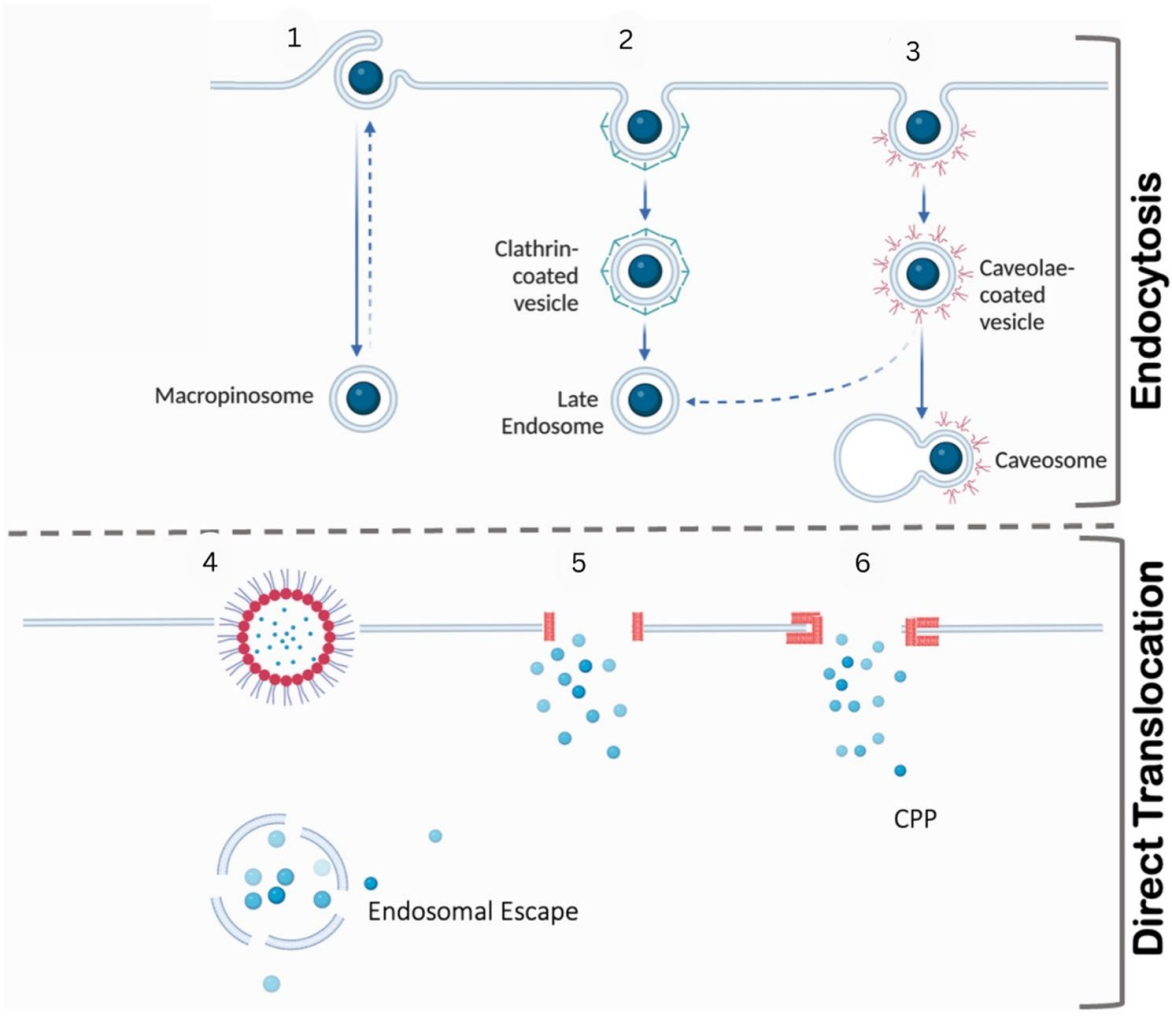
Intracellular delivery systems within cells that includes overview of protein trafficking pathways. These pathways include a variety of methods, including endocytosis, which is elaborated in multiple steps. (1) Macropinocytosis; (2) Clathrin-mediated endocytosis; and (3) Caveolae-mediated endocytosis. Direct translocation mechanisms are also presented, including (4) the Inverted micelle model, (5) the Pore model, and (6) the Carpet model

Endocytosis is a fundamental cellular process through which eukaryotic cells internalize macromolecules by plasma membrane invagination. It plays essential roles in signal transduction, membrane homeostasis, mitosis, adhesion, lipid regulation, motility, and cell morphogenesis [80, 81]. There are a variety of pathways by which endocytosis mediates the uptake of extracellular cargo [82] (Fig. 2).

Endocytic process called macropinocytosis corresponds to the nonspecific ingestion of extracellular media, resulting in large vesicles that are named macrophinosomes (Fig. 2(1)), macropinocytosis is highly relevant during nutrient uptake as well checkpoint for immune surveillance, it allows the transfer of solutes or antigen inside to out cell [80]. Clathrin-mediated endocytosis, probably represents the most studied form of all and is defined by vesicles with clathrin coats which internalize certain cargoes, i.e. former cell surface receptors or nutrients (Fig. 2(2)) [82]. Caveolae-mediated endocytosis is another important endocytic pathway in which the plasma membrane invaginates to yield small, flask-shaped structures enriched with caveolin proteins. It is essential to lipid metabolism, signal transduction and the entry of some (but not all) pathogens (Fig. 2(3)) [1].

### Direct translocation

In addition to these classical routes of endocytosis, there are also direct translocation mechanisms for various molecules that traverse across the plasma membrane without vesicle formation [83] (Fig. 2). As in the inverted micelle model, some molecules aggregate in a manner that causes plasma membranes to become grouped into an arrangement similar as to what is seen on antimicrobial peptide surfaces; consequently this results their ability of passing through and entering cells (Fig. 2(4)) [84]. Original hypotheses set forth that protein-induced pores were formed in the plasma membrane thus allowing diffusion of molecules into the cytoplasm (pore model) (Fig. 2(5)) [85]. The model assumes that the membrane remains partially intact even after the formation of the pores and thus, allows for a selective permeability of the molecules (Fig. 2(6)) [85]. This mechanism has been observed in systems ranging from antimicrobial peptides to therapeutic protein delivery systems and certain drug carriers that use the pores to ferry drugs inside the cell without necessarily killing the cell.

The carpet model describes a mechanism whereby amphiphilic molecules such as peptides or lipid disrupting agents attach themselves to the membrane surface extensively such that they form a carpet-like layer [84]. This interaction causes the lipid bilayer structure to break down to either full disintegration or the formation of micellar structures that ferry molecules into the cell. The carpet model is different from the pore model in that it does not involve pore formation but rather a minimal number of amphiphilic molecules that are required to bring about the membrane destabilization (Fig. 2(6)) [86]. This mechanism is of particular importance for highly amphiphilic molecules, which are characterized by a strong tendency to disrupt the membrane and, therefore, can be used for the cellular delivery of hydrophobic drugs and protein complexes. These pathways, therefore, demonstrate the sophisticated yet distinct roles of cellular internalization [86].

### Invasin-mediated uptake

**Fig. 3:**
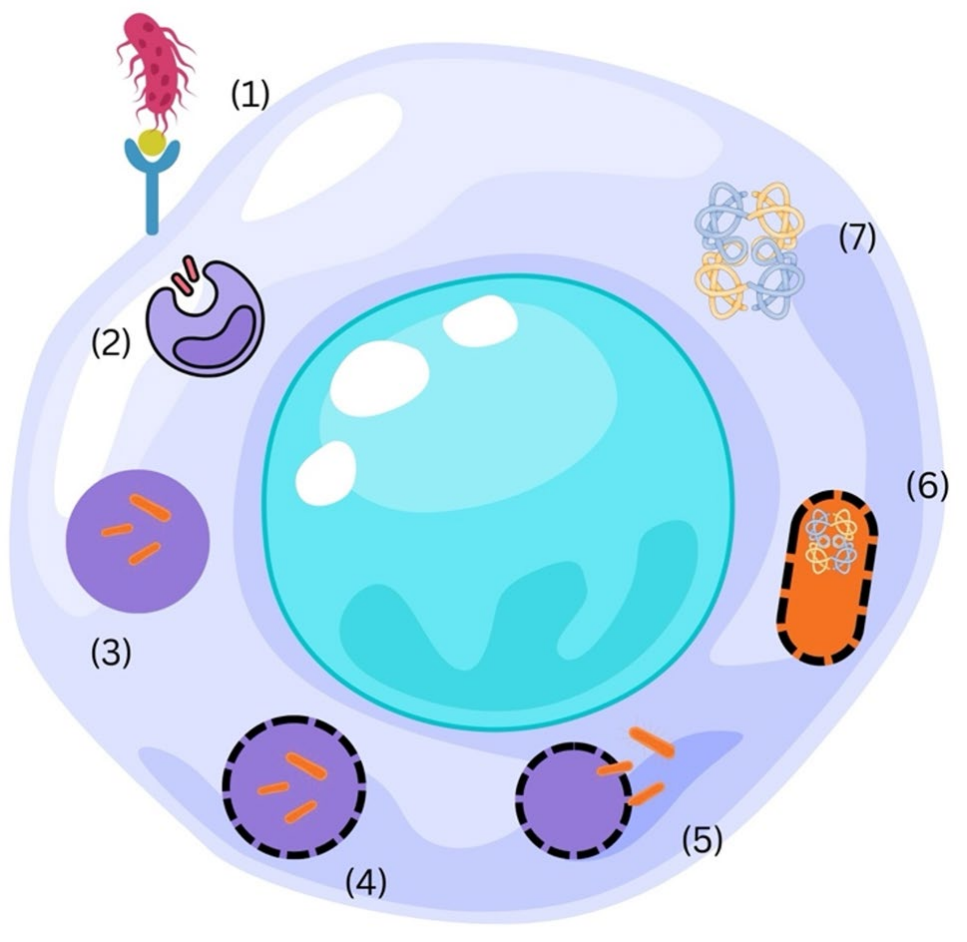
Schematic representation of the invasin-mediated uptake pathway enabling *E. coli* entry into mammalian cells. (1) Engineered *E. coli* binds to β-integrin receptors on the host cell surface via invasin. (2) The interaction triggers receptor-mediated endocytosis and vesicular internalization of the bacterium. (3) *Listeriolysin O* facilitates escape from the endosomal compartment by disrupting the vesicle membrane. (4) The bacterium is released into the host cytoplasm. (5) Controlled lysis of the engineered *E. coli* enables intracellular release of the therapeutic protein cargo. (6) The delivered protein subsequently becomes available within the host cell cytoplasm

Invasin-mediated internalization is a powerful approach in protein engineering of *E. coli* for direct delivery into mammalian cells as shown below in Fig. 3 [87]. Invasin is a β₁-integrin–binding protein from *Yersinia pseudotuberculosis* that provides a “zipper-type” entry mechanism to enable the invasion of normally non-phagocytic cells by non-pathogenic *E. coli* [88, 89]. Early landmark studies have demonstrated that *E. coli* engineered with the *inv* gene can deliver functional heterologous proteins [90], such as β-lactamase or GFP, into the cytosol when co-expressed with *Listeriolysin O* (LLO), mediating endosomal escape [11, 91, 92].

Recent progress has significantly enhanced the utility of invasin-based delivery platforms. In 2021, it was shown that host factors, such as MUC1, promote β₁-integrin clustering and thereby enhance the internalization efficiency of invasin-expressing engineered *E. coli*, thus increasing the amount of bacterial protein cargo reaching host cells [93]. Newer synthetic biology approaches (2020–2024) have coupled invasin to secretion or release modules, including Type III secretion systems, autotransporters, and OMVs, for targeted delivery of functional proteins-including transcription factors, immunomodulators, and therapeutic enzymes-following bacterial uptake [35, 59, 94–96]. Cumulatively, these advances indicate that invasin-mediated uptake is not only a robust method for enhancing host cell entry but also a cornerstone mechanism enabling *E. coli* to function effectively as an intracellular protein delivery vector.

### Challenges and considerations in intracellular protein delivery

The ability to overcome challenges such as low efficiency of internalization and rapid release into the cytosol without being trapped in endosomes is still crucial [97]. Current research explores diverse delivery tools, including protein conjugation with cell-penetrating vectors and multifunctional chimeric peptides designed for enhanced cellular uptake and endosomal escape [98]. The general assumption is that nanocarriers are transported to the cytoplasm through the endosomes [99, 100], when the endosomes are transformed into lysosomes the environment becomes more acidic and proteases are activated leading to protein degradation.

However, delivering functional proteins into cells inherently faces the barrier that most proteins are large, hydrophilic macromolecules, unable to cross the plasma membrane easily [101]. For this, a carrier system has to be used to assist the transport of these proteins intracellularly while preserving protein stability and activity. Protein surfaces are heterogeneous and contain cationic, anionic, and hydrophobic regions which may affect how proteins interact with various delivery platforms [101].

Covalent attachment is one of the numerous ways to associate proteins with carriers, and it is not always necessary. Covalent conjugation offers controlled orientation and stability but also has its own challenges, with restricted reactive residues and potential disruption of protein folding or function [1]. In contrast, several delivery systems (i.e., polymeric nanoparticles, liposomes, and protein-based nanocarriers) can encapsulate proteins non-covalently by means of electrostatic adsorption, hydrophobic interactions, or physical entrapment without the chemical modification of the protein [102].

Alternatively, CPPs or trafficking peptides are genetically fused to proteins through recombinant cloning, providing receptor-mediated uptake or direct membrane interaction without the need for chemical linkage [103]. Safe and effective protein delivery approaches therefore rely on mechanisms such as endocytosis followed by endosomal escape or, in some cases, direct cytosolic translocation facilitated by membrane-active peptides or bacterial secretion systems. These strategies collectively enable the delivery of functional proteins into the cytoplasm while limiting structural perturbation or loss of activity [102, 104, 105].

Effective protein transduction across cellular barriers can be influenced by host immune responses. As with many conventional protein therapeutics, proteins delivered into or onto cells may be recognized and neutralized by the immune system, which can limit their stability and functional activity [106]. This immunogenicity challenge is therefore not unique to intracellular protein delivery but represents a broader consideration in the development of protein-based therapies. To mitigate these effects, several strategies have been implemented; the engineering of delivery systems to have stealth properties such as altering the surface properties to reduce immunogenicity and increase the circulation time [107]. Moreover, selection of appropriate biomaterials and fabrication of delivery systems that can evade the immune system are also vital in the course of this process. It is crucial to understand the immune context in protein delivery in order to design effective strategies for increasing the stability and biological activity of delivered proteins [108].

One of the advancement has been reported, where *E. coli* has been transformed to act as a bacterial delivery system; this is because it has some advantages that could help overcome the previous challenges. Genetically engineered *E. coli* is capable of producing and secreting therapeutic proteins directly at the target site and therefore eliminates the need for external delivery vectors which increases the availability of the delivered proteins [19]. Furthermore, the surface antigens can be changed in order to hide the cell from the immune system by altering the antigenicity of the surface [109]. *E. coli* can also increase the specificity of delivery by displaying certain surface ligands that will only recognize the target cells and no other cells [110]. Furthermore, *E. coli* can deliver several proteins or therapeutic agents at once, which is harder to achieve with traditional drug-delivery systems but results in greater overall efficacy during therapy [111]. These advantages demonstrate the potential of *E. coli* as a single tool for solving the problems of intracellular protein delivery.

## Outer membrane vesicles (OMVs) in protein delivery

### Engineering OMVs for protein delivery

Derived from the outer membrane of gram-negative bacteria, Outer Membrane Vesicles (OMVs) contain lipids, proteins, peptidoglycan, and nucleic acids in varying amounts [112, 113]. Among its principal roles, OMVs carry these molecules to other cells, whether host or bacterial cells [114]. Engineered OMVs represent the most versatile nanocarriers for protein delivery into mammalian cells, due to their inherent stability, immunomodulatory properties, and natural ability to fuse with or be internalized by host cells [115]. Cargo can be loaded via passive encapsulation, surface display, or active targeting motifs. The fusion of proteins with OMV-targeting sequences, such as the *ClyA* scaffold, AIDA-I autotransporter, or *Lpp-OmpA* fusion tag, efficiently drives the localization of heterologous proteins into or onto OMVs [116].

These systems have lately been further improved through the use of synthetic biology methodologies, which include, among others, programmable OMV loading by means of inducible promoters or modular secretion tags [117]. Other recent work (2021–2024) includes detoxified OMVs, generated by deleting *msbB* or *lpxM* to reduce LPS endotoxicity, and hypervesiculating strains created by mutating *tolA*,* tolR*,* nlpI*, or *degP*, which increase OMV yield and cargo load significantly [118, 119]. Collectively, all these strategies are enabling the production of safer, more efficient, and better-suited OMVs for application in the delivery of therapeutic proteins.

### Uptake pathways of OMVs

Uptake of OMVs into mammalian cells occurs by a variety of endocytic and non-endocytic pathways [120]. The pathway chosen has a significant impact on the way cargo is released and also on its intracellular trafficking. The major pathways followed are clathrin-mediated endocytosis, caveolin-mediated endocytosis, and micropinocytosis [121]. The choice between uptake pathways depends on the size of the OMV, the composition of LPS, surface proteins, and also on cell type [122].

Recent studies have demonstrated the potential of engineered OMVs, carrying either modified LPS or decorated with host-targeting ligands such as integrin-binding peptides or TLR ligands, to alter the route of uptake [123]. For example, they can result in increased caveolin-mediated uptake to enhance cytosolic delivery. Engineering of OMVs with fusogenic peptides, pH-responsive domains, or endosomal escape elements such as HA2, GALA, or H5WYG has been reported during 2022–2023 to exhibit an improved release of proteins into the cytosol following endocytosis [124]. Such a wide variety of uptake mechanisms makes OMVs strong contenders for effective delivery to the cytosol, endosomal compartments, or antigen-presenting pathways.

### Therapeutic applications of engineered OMVs

Engineered OMVs are increasingly used as platforms for protein-based therapeutics, vaccines, and intracellular delivery systems [115]. In cancer therapy, OMVs loaded with tumor-associated antigens, immune-modulating proteins, or cytokines have shown potent anti-tumor responses in preclinical models (2020–2024) [125]. For example, OMVs carrying PD-1 nanobodies, interleukin cargo, or ClyA-fused tumor antigens triggered robust T-cell activation and tumor regression in murine models [126]. In infectious disease applications, OMVs displaying viral or bacterial antigens-including SARS-CoV-2 RBD, influenza HA, and *Listeria* antigens-have demonstrated strong protective immunity, leading to several OMV-based vaccine candidates progressing to preclinical and early clinical evaluation [127]. Therapeutically, OMVs have been used for intracellular enzyme replacement, such as OMV-mediated delivery of β-lactamase, Cas9 ribonucleoproteins, or anti-inflammatory proteins into target cells [112]. Collectively, these examples highlight the rapidly expanding therapeutic landscape of engineered OMVs and their potential as safe, modular, and scalable delivery vehicles for intracellular protein therapeutics.

### Applications of *E. coli* vectors for protein delivery

*E. coli* strains have been genetically engineered to deliver high specificity protein payloads in-situ at the sites of disease with high specificity [111]. Using synthetic biology methodologies, pathogenic lab and non-pathogenic human *E. coli* isolates have been engineered to harbor a Type III secretion apparatus that permits regulated or constitutive secretion of therapeutic proteins into cells [128]. A representative example of advanced chassis engineering is the study in which nonpathogenic *E. coli* was equipped with key structural and regulatory components of the Shigella Mxi–Spa Type III Secretion System (T3SS) [57, 59]. The authors introduced essential proteins such as MxiG, MxiJ, MxiD (basal body/outer-membrane ring), Spa proteins (export apparatus and ATPase complex), and regulatory elements including MxiE and the chaperone IpgC, enabling assembly of a functional secretion needle in *E. coli*, by fusing cargos to the N-terminal secretion signals of IpaB or OspF, the engineered strain was able to translocate diverse proteins into the mammalian cytosol, including enzymatic reporters like β-lactamase and effectors that modulated intracellular pathways such as MAPK and NF-κB signaling [57].

**Fig. 4:**
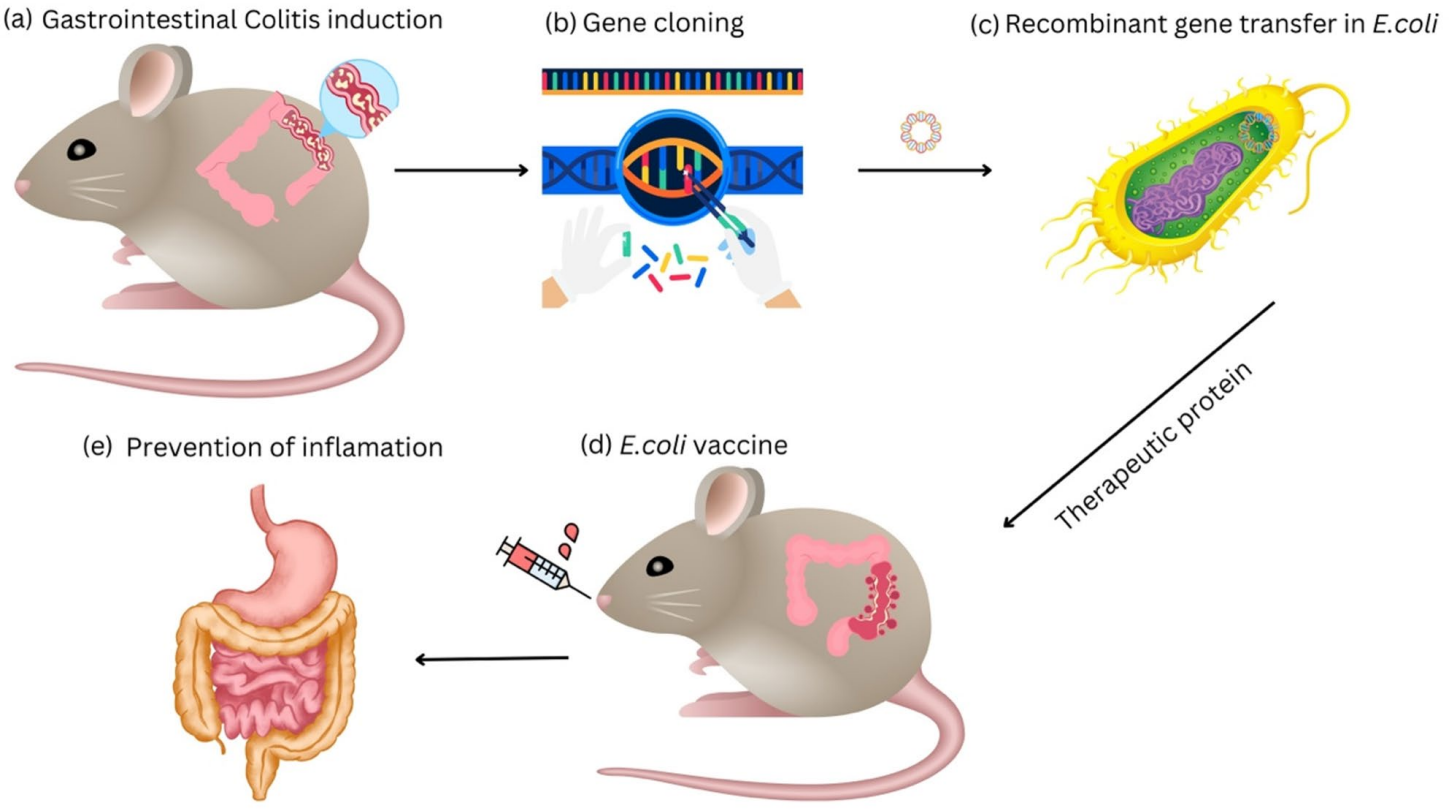
Schematic representation of Colitis treatment using *E. coli* as a therapeutic carrier: **a** Induction of Colitis: 6.5 + CD4+ transgenic T cells enriched from spleen and MLN of TCR-HA mice were injected into RAG1−/− mice, **b** Gene cloning: The gene encoding the therapeutic enzyme is cloned into a bacterial plasmid, **c** Bacterial transformation: The transformed plasmid enters the *E. coli*. Under appropriate induction, the bacteria express the pharmaceutical compound therapeutic antigen, **d** Targeted treatment: The engineered *E. coli*, now with the biotherapeutic antigen, is injected directly into the gastrointestinal tract, and **e** to mitigate inflammation and facilitate healing

Another example is *E. coli* Nissle 1917 (EcN), a live carrier for targeted delivery of recombinant proteins to the intestinal mucosa [129]. A genetically modified EcN strain, already approved by Dutch authorities for experimental treatment of inflammatory bowel disease [130], was further engineered to express therapeutic proteins and tested in a highly sensitive CD4⁺ T-cell immunological model [129] (Fig. 4). As shown in Fig. 4, the recombinant EcN did not interfere with T-cell migration, activation, clonal expansion, or tolerance mechanisms, neither in healthy mice nor in mice with acute colitis, which indicates that this approach of localized antigen or protein delivery will not lead to exacerbation of inflammation or autoimmunity [129]. Due to its potent colonization and excellent safety profile under both normal and diseased conditions, EcN represents a promising microbial chassis for gut-focused in situ synthesis of therapeutic molecules [129]. This system illustrates how engineered *E. coli* can serve as a platform for targeted intracellular antigen/protein delivery.

These approaches are consistent with the numerous functions of *E. coli* in protein delivery including in vaccine development and oral protein therapy [131]. For example, *E. coli* has been engineered to deliver anticancer proteins directly to tumors in murine models [132] (Table 1)). Research by [133] reported the use of engineered *E. coli* strains that can recognize and respond to inflammatory signals in the gut, thereby targeting the delivery of therapeutic proteins to treat inflammation in IBD models. Similarly, *E. coli* strains have been developed to identify and eliminate *P. aeruginosa*, a pathogen that is most especially associated with burn wound infections [134]. These findings underscore the significance of *E. coli* vectors in the development of site-specific drug delivery systems, which can significantly improve precision medicine [135].

In addition, live attenuated vaccine vector *E. coli* has also given positive outcome [136]. proposed *E. coli* based vaccines for diarrheal diseases associated with ETEC and the immunity was promising. In a similar fashion [137], investigated the immunogenicity of live attenuated *E. coli* expressing *UreB* of *Helicobacter pylori* and the results showed positive mucosal immune response. These studies show how *E. coli* vectors can be easily tailored to meet the needs of different infectious diseases through vaccination. Moreover, *E. coli* vectors have been extensively applied for the development of vaccines for infectious diseases and cancer immunotherapy as summarized in Table 1. In oncology, *E. coli* has been engineered to express tumor associated antigens, such as melanoma associated proteins, and is therefore a potential cancer immunotherapeutic agent [138].

Thus, the above listed achievements it is possible to state that *E. coli* remains a versatile and large-scale system for both prophylactic and therapeutic vaccines, and thus is an important player in the new vaccinology.

**Table 1:** Represents research utilizing bioengineered *E. coli* as drug delivery platforms

E. coli strains	Target of usage	Function	References
*E. coli* Nissle 1917	Delivery of p53 and Tum-5 to solid tumors	Cancer	[139]
*E. coli* Nissle 1917	Produce 3-hydroxybutyrate	Colitis	[140]
*E. coli* Nissle 1917	Delivery of matrix-tethered therapeutic domains	Gut	[141]
*E. coli K-12* (engineered invasive strain	Recombinant therapeutic or reporter proteins (varies)	Delivery into mammalian cells through engineered invasion machinery	[142]
*E. coli* K-12	β-lactamase and other recombinant proteins	Cytosolic delivery into macrophages for immune-modulation or protein replacement	[143]
*E. coli* (wild-type/modified for OMV production)	OMV-encapsulated proteins or drugs	Transdermal penetration & tumor-targeted delivery using vesicles	[144]
*E. coli* Nissle 1917	Intracellular recombinant proteins (various)	Autolysis-based release of cargo for extracellular delivery	[145]
*E. coli* (recombinant expression strain)	HIV-1 Nef protein (Tat-tagged)		[146]
Non-pathogenic *E. coli* (display-engineered)	IL-18 mutein on bacterial surface	Local immune activation in tumor microenvironment to enhance CAR-NK cell activity	[147]
*E. coli* O83		OMVs containing immunomodulatory proteins	[148]
*E. coli* Nissle 1917	Chemotherapeutic drugs (loaded into minicells)	Targeted drug delivery to hypoxic regions of tumors	[149]

## Challenges and future directions

### Immunogenicity and safety concerns

The deployment of *E. coli* as vectors for protein delivery into humans introduces intricate challenges and prompts thoughtful considerations for immunogenicity and safety [150]. Immunogenic responses, a consequential concern, may emanate from the host’s recognition of *E. coli*-derived human recombinant proteins, Potentially compromising the intended therapeutic outcome, residual bacterial constituents in delivered proteins pose a significant challenge [107]. For instance, Lipopolysaccharides (LPS) and endotoxins associated with *E. coli* cell walls have a strong immune stimulatory effect [150] and these components can enhance the likelihood of immunogenic reactions, thus jeopardising the safety and efficacy of therapeutic applications. Purification is strict to avoid the presence of other bacterial components like LPS, flagellar proteins and other immunogenic debris.

Recent efforts have increasingly focused on “deimmunizing” *E. coli* through genetic engineering [151, 152]. These strategies include modifying or deleting genes involved in LPS biosynthesis to reduce the endotoxic activity of lipid A, attenuating surface-associated immune triggers, and redesigning outer membrane structures to minimize host recognition [153, 154]. Such approaches offer a more direct means of improving the safety and tolerability of *E. coli*-based protein delivery systems by intrinsically reducing their immunogenic potential [154]. Affinity chromatography, ultrafiltration and endotoxin removal resins are some of the methods used to obtain the desired purity [69]. Further, the safety of the vectors should be improved by enhancing the above-mentioned purification techniques and finding new delivery systems based on nanocarriers and smart biomaterials to reduce immunogenicity and enhance the therapeutic effect [155].

### *E. coli’s* efficiency in protein expression and its delivery into cells

In the field of protein delivery using *E. coli* vectors, *E. coli’s* human recombinant protein expression yield and its efficiency in delivery to the cells are critical challenges and future considerations. However, *E. coli* has its advantages in terms of cost and ease of genetic manipulation [34]. Problem such as low protein expression, incomplete delivery or ineffective release of the material inside the target cells may affect the therapeutic efficacy. It is crucial to maintain a good control on the protein production in order to increase the delivery efficiency and to avoid improper folding and post translational modifications of the proteins [150]. Future work should be directed towards improving the expression systems, delivery systems and finding new ways to produce more quantities of proteins with higher efficiency so that *E. coli* can be used more effectively as a vector for protein delivery in various biomedical applications [156].

### Ethical considerations

This review article also focuses on the regulatory frameworks that supports the biopharmaceutical productions using *E. coli* are safe, pure and can be reproduced [157]. In the case of *E. coli*-based protein delivery systems, the need to comply with these regulations is important to avoid the consequences or effects that may be adverse. It is crucial to maintain the balance between the technical aspects and the regulatory aspects in order to comprehend the dynamics of biopharmaceutical development.

Ethical issues are also important and particularly so with regard to the environmental release of genetically modified *E. coli* strains [158]. pointed out that there is a need to ensure that risk analysis is well carried out and enforcement of policies that help in avoiding the risks that are associated with ecological and human health effects. For example, investigation of *E. coli* vectors for on-site drug making needs to determine how well the vectors can be contained to avoid their release into the environment. It is therefore crucial to maintain the dialogue between regulatory bodies, research institutions, and ethical committees in order to set up clear rules which define the permissible scope of *E. coli*-based protein delivery [159]. These efforts guarantee that research and applications are carried out with scientific rigor and in conformance with the societal values, so as to sustain the public confidence in the technology.

### Safety considerations, containment strategies, and regulatory requirements

Auxotrophy-based containment continues to be one of the most robust and widely employed biosafety improvement strategies for engineered *E. coli* [160]. Auxotrophic mutants, such as DAP-dependent, thymidine-dependent, or synthetic amino acid-dependent strains, can survive only in supplemented conditions and thus avoid uncontrolled growth in natural environments [160, 161]. Recent demonstrations of such approaches in clinically intended strains include DAP-auxotrophic *E. coli* Nissle platforms developed for cancer therapy and genomically recoded *E. coli* dependent on noncanonical amino acids, which completely ablated escape events [11, 162].

Further, while *E. coli* Nissle 1917 is widely used owing to its established probiotic [163–165] and GRAS-like safety profile, EcN carries the pks genomic island responsible for colibactin production, which poses concerns relating to genotoxicity [165]. Several recent studies have confirmed context-dependent DNA damage associated with pks-positive strains [166], and many therapeutic platforms have since adopted pks-deleted EcN derivatives to mitigate this risk [167]. Finally, engineering approaches toward the use of *E. coli* for the delivery of therapeutic proteins have to be performed within well-defined regulatory frameworks, such as FDA guidance on Live Biotherapeutic Products, NIH recombinant DNA guidelines, EMA LBP requirements, and OECD GMO principles on risk assessment [168, 169]. Taken together, these approaches focus on strain characterization, validation of biocontainment, evaluation of horizontal gene transfer, deletion of virulence determinants, and environmental safety.

### Emerging technologies and innovations: synthetic biology approaches and hybrid vector systems

The advent of synthetic biology has opened new frontiers in *E. coli*-mediated protein delivery by enabling the design of sophisticated genetic circuits and hybrid vector systems. Advances in gene editing and modular design principles have facilitated the creation of bespoke *E. coli* systems for targeted and efficient protein delivery [19]. For example, engineered *E. coli* strains have been programmed with synthetic circuits that produce therapeutic proteins only in response to specific biomarkers, such as tumor-associated antigens, which enhances precision in cancer therapy and reduces off-target effects [170]. However, these innovations introduce challenges related to predictability and stability. The development of synthetic systems demands careful attention to unintended consequences, such as metabolic burden or genetic instability, which can compromise system performance. Computational modelling and iterative testing can mitigate these risks by improving the reliability of synthetic gene networks [171].

There are several counter examples that could be given to this. A specific case is the optimization of the promoter sequences and the ribosome binding sites to obtain the same level of protein expression across different environmental conditions to make the production of the therapeutic protein more stable [170]. Moreover, the problems of the scalability and the reproducibility are critical for the effective implementation of the synthetic biology knowledge into practice.

There are promising strategies for improving the delivery efficiency and therapeutic output through hybrid delivery systems, which are *E. coli* vectors combined with other platforms. For example is the application of *E. coli* for the production of therapeutic proteins in conjunction with polymeric nanoparticles for the delivery of small-molecule drugs, which led to enhanced therapeutic outcome in preclinical models of inflammation [172]. However, these hybrid systems are rather complicated and thus require further investigation of vector compatibility and biochemical interactions to combine them optimally, the standardization of the hybrid approaches is very important for their efficacy and reproducibility in different applications. The development of standard operating procedures for constructing and testing hybrid vectors has helped to expand their use in industrial-scale biopharmaceutical manufacturing [173].

Some ethical and environmental issues are connected with the application of hybrid systems, including the potential of releasing genetically modified organisms. To address these challenges, comprehensive risk assessments and clear regulatory mechanisms are critical [157]. Future work on *E. coli*-based protein delivery should therefore focus on the improvement of design principles, the reduction of system unpredictability, and the definition of standard operating procedures. In order to harness these innovations to their fullest potential, while preserving scientific robustness, efficacy and ethical integrity, interdisciplinary collaborations will be essential.

## Conclusion

Engineering *E. coli* as a programmable vehicle for intracellular protein delivery is rapidly advancing, providing an important link between laboratory research and clinical translation [142]. Researchers have harnessed the natural machinery of the bacterium to develop flexible, cost-effective platforms enabling the production and delivery of therapeutic proteins with great precision [11, 149]. In addition, advances in synthetic gene circuits, including hypoxia-inducible promoters, stimulus-responsive lysis systems, and clinically relevant regulatory modules, have provided similarly accurate, context-dependent control over protein expression and release [50].

Notwithstanding these developments, several obstacles still need to be resolved before the adoption of *E. coli*-based systems into clinical practice can become widespread. The main limitations include immunogenicity, off-target effects, and scalability [142, 168]. These challenges are being mitigated through advances in synthetic biology, more efficient purification strategies, and better biocontainment approaches, which all contribute to enhanced biosafety, reproducibility, and therapeutic control [161].

The full realization of *E. coli* as a therapeutic delivery agent will depend on strong interdisciplinary collaboration among molecular biologists, bioengineers, clinicians, and regulatory experts. As technological innovation continues, strategically engineered *E. coli* strains are set to assume key roles as robust and versatile therapeutic systems. Ongoing optimization efforts will further improve their safety, specificity, and efficacy, thereby setting the stage for future translational breakthroughs in medicine and biotechnology.

The full realization of *E. coli* as a therapeutic delivery agent will depend on strong interdisciplinary collaboration among molecular biologists, bioengineers, clinicians, and regulatory experts. As technological innovation continues, strategically engineered *E. coli* strains are set to assume key roles as robust and versatile therapeutic systems. Ongoing optimization efforts will further improve their safety, specificity, and efficacy, thereby setting the stage for future translational breakthroughs in medicine and biotechnology.

## Data Availability

No new data were created or analysed in this study. Data sharing is not applicable to this article.
